# An evaluation of the impact of a cancer support specialist service on families of children with cancer and the multidisciplinary team in a children’s health service in Ireland

**DOI:** 10.1177/13674935241312722

**Published:** 2025-01-06

**Authors:** Maryanne Murphy, Hugh Fulham-McQuillan, Agnes Higgins, Maria Brenner

**Affiliations:** 1 School of Nursing and Midwifery, Trinity College Dublin, Dublin, Ireland; 2 School of Nursing, Midwifery and Health Systems, University College Dublin, Dublin, Ireland; 3 Department of Mental Health, School of Nursing and Midwifery 8809 Trinity College Dublin, Dublin, Ireland; 4 Department of Children’s Nursing, UCD School of Nursing, Midwifery and Health Systems, University College Dublin, Dublin, Ireland

**Keywords:** Childhood cancer, informal psychological support, service evaluation, parental needs

## Abstract

The onset of childhood cancer is sudden and unexpected, and the effect on the family unit can be enormous as they embark on a major life transition. Families of children with cancer have a high level of psychosocial needs due to the many challenges they may face during their child’s cancer journey. Previous research indicates that the current healthcare system does not always meet these needs. This qualitative descriptive study aimed to explore the impact of a new Cancer Support Specialist Service from the perspective of the families and the multidisciplinary team. Data were collected using semi-structured one-to-one interviews. The impact on the family was increased emotional, practical, informational, and navigational support. The impact on the MDT included freeing up time for the clinical component of their work, decreasing worry that unmet needs for parents were not being addressed, and increasing access to timely flexible support for families. The knowledge advanced by this study can inform future planning of the Cancer Support Specialist Service.

## Introduction

Learning that a child has been diagnosed with cancer can be the most distressing experiences of a parent’s life (Carlsson et al., 2019). Family life experiences disruption, and everyday life of families changes as they confront the reality of childhood cancer (Bilodeau et al., 2018; Long and Marsland, 2011).

Providing adequate support to families can enhance a family’s overall experience of a childhood cancer journey while alleviating some of the associated stress (Melesse et al., 2022). Families require a universal level of psychosocial support which includes assistance in navigating available statutory health services as well as non-statutory services such as charities (Delemere et al., 2023).

Previous research (Delemere et al., 2023; Kerr et al., 2007), including phase one of this project (Martin et al., 2017), highlighted that current healthcare systems do not always meet the multiple needs of families on a childhood cancer journey. With this in mind, and with a shared vision of addressing the informal psychosocial needs of families of children, a Cancer Support Specialist Service (CSSS) was implemented in a hospital and community setting in Ireland. The service included a specific Cancer Support Specialist (CSS), and requirements for role holders of CSS posts were to have a recognised professional qualification in one of the following areas: Social Work, Community Youth work, Play Therapy, Nursing, A Therapeutic discipline, or other relevant professional qualification. The CSSS arose from a partnership between the Katie Nugent Fund (KNF), the Cancer Fund for Children (CFFC), and Children’s Health Ireland (CHI) at Crumlin Hospital, Dublin. At the time of this study’s completion, the CSSS comprised of five CSS roles. The first post based in a ward setting was in place six months before the beginning of this study. Four community-based CSS roles were implemented during the study period (Oct 2022–Dec 2023). The CSS role holders were to provide generic informal psychosocial support to families and, in doing so, provide auxiliary support to the MDT on the children’s oncology ward.

## Aim

To evaluate the impact of a Cancer Support Specialist Service from the perspective of families and the multidisciplinary team (including the CSSs).

## Methods

A qualitative descriptive design was used in this study to evaluate the impact of the role on families and MDT members. Given an emphasis on staying close to the data and due to its flexibility, a qualitative descriptive design is deemed a useful method for giving voice to recipients of care, family members, and healthcare staff (Doyle et al., 2020).

### Recruitment

A purposive sampling strategy was used to recruit participants in this study. Inclusion criteria were families who had or were currently using the new CSSS. MDT members, CSS role holders, and members of the study’s steering group were also included. Exclusion criteria were families who had not used the service and MDT members who had not worked with the service.

## Data collection

Participants were recruited from October 2022 to December 2023. The target population were parents, CSS role holders, members of the MDT, and key members of the study’s steering group, using one-to-one semi-structured interviews. The research team (HFMcQ, AH, and MM) developed an interview guide with questions to evaluate the impact of the CSSS on families and the MDT.

## Data analysis

Interviews were transcribed verbatim, pseudoanonymised, and uploaded to NVivo 12 (QSR International Pty Ltd, 2018) prior to data analysis. Pseudonyms were used throughout the analysis and in preparation of this manuscript to avoid inadvertently identifying participants. The first round of analysis employed Braun and Clarke’s (2022) six-step approach to data analysis, which involved coding data in each interview into subcodes. The second round involved coding subcodes into under two over-arching codes, namely, the impact of the CSSS on the MDT and the impact of the CSSS on families. Two members of the research team independently coded over 50% of the interviews (HFMcQ and MM) and came together with the third researcher (AH) to discuss codes, subcodes, and supporting data. At this stage, codes were compared and collapsed and a final list of codes and subcodes were agreed. To ensure interrater reliability, each researcher doubled-coded a randomly selected 10% of the other’s data.

## Ethical issues

This study was conducted in accordance with the Helsinki Declaration. Ethical approval was obtained from an ethics committee in Children’s Health Ireland (approval no. REC-046-21) and from the Faculty of Health Sciences Ethics Committee, Trinity College Dublin (approval no. 220603). All ethical guidelines for human research were followed. Participants gave informed written consent. Participants were reminded that they could withdraw without explanation. Transcripts were anonymised. Pseudonyms are used for participant quotations.

## Quality and rigour

A number of strategies used to ensure quality and rigour of the study (Supplement file one).

## Findings

Thirty-four interviews were completed with 31 (*n* = 31) participants, as three participants were interviewed twice. Repeat interviews were performed to gain a perspective on the service as it was evolving. Twenty-three participants were members of the MDT (including CSSs and a member of the steering group), and eight participants were family members. The majority of participants were female (28) and the remaining three participants were male. Further details can be seen in Table 1.

**Table 1.** Participant profile.

	Number of participants	Discipline/role/relation to child	Other relevant information
MDT members	23	Nurses, social workers, consultant oncologists, dieticians, psychologists, steering group member, and CSSs (hospital = 1 and community = 3)	Interviews completed = 26 (3 MDT members were interviewed twice)
Family member	8	Mother = 7	Parents of
			2-year-old boy
		Father = 1	2-year-old boy
			13-year-old girl
			13-year-old boy
			14-year-old boy
			16-year-old girl
			16-year-old girl
			16-year-old boy

• MDT, multidisciplinary team members. • CSS, Cancer Support Specialist.

Following data analysis, two themes with relevant subthemes were identified. These were Impact of the CSSS on Families, and Impact of the CSSS on the MDT.

## Impact of the CSSS on families

Within this theme, five subthemes were identified, highlighting differing types of support provided by the CSSS to families. These were Relational Support, Mediation Support-Bridging the Gap, Navigational Support, Respite Support for Parents, and Support to Siblings.

### Relational Support

Parents reported overwhelmingly on the positive impact of relational support offered by CSSs at times of immense emotional distress. Words such as ‘*fantastic’*, *‘amazing’*, *‘willing*,*’ ‘caring*,*’ and ‘the rock’* permeated the interviews. Parents valued the idea of having a CSS that was solely there to support them in their childhood cancer journey and was described by a parent as ‘*just being there’*. (Amir-parent)

*‘… I don’t know how I can put it into words, to be honest, it's very hard but*

*[the CSS] make[s] a difficult journey much easier’.* (Caroline-parent)

The ‘ready availability’ of a CSS was mentioned by parents as a key benefit. Parents valued that CSSs were proactive in offering help:

‘*it's just nice to have someone just to check in too’.* (Kate-parent)

As the sole focus of the CSSs was to support families, parents felt assured that role holders did not need to rush off to deal with other issues, and could stay with them when needed:

*‘I think it just brought a bit of lightness to the day, you know, knowing that there's someone that you can just kind of sit and have a chat [with]and they don’t have to run off to somebody else… So, I think it’s just that kind of personal touch, which is really important, you know.…As in we could go off and have a coffee and kind of a more relaxed chat I suppose, [the CSS] really [was not] being pulled in all directions’.* (Ellen-parent)

Some parents appreciated being able to chat with CSSs in a ‘normal’ way, and not have to feel they were in a formal therapy context:

*‘…not necessarily counselling, but just to have a chat with somebody who kind of gets *it*...’.* (Daniela-parent)

*‘And it’s nice as well because [the CSS] has a clinical background I can chat to [The CSS] as well, do you know what I mean? [The CSS] understands what I’m talking about, you know, to depths that other people don’t. So, if I’m telling [the CSS] he’s [child’s name] going through X, Y, or Z [the CSS] just knows’*. (Daniela-parent)

Some parents spoke of an additional benefit of the CSS’s support over support from family or friends as there was no expectation of reciprocity. Similarly, parents also felt they did not have to worry about protecting the CSS from emotional pain and trauma that they were going through:

‘[The CSS is] someone to talk to when you mightn’t want to, you get sick of ringing your family, I think your family is sick listening to you, you know (laughs), you don’t wanna be the bringer of bad news all the time and it's nice to have someone that can *just listen, you know’.* (Caroline-parent)

An MDT member also spoke of the benefit of the CSS’s support over support from the MDT.

‘You know sometimes they will tell the cancer support specialist things that they *won’t necessarily tell us’*. (Isla-MDTM)

Members of the MDT also spoke of the positive impact and additional support offered to parents by the CSS particularly at times when other services may not be available.

*MDTM (Multidisciplinary Team Member)

*‘So they kind of almost they bridge the gap between the clinical and the more informal relationships that the families have’.* (Olivia-MDTM).

‘*I think again being able to plug that hole or the gap in the social support piece for parents is really crucial*… *And with the best will in the world we do it social workers do it, the physios do it, the play therapist do it, but we don’t always have the time…’*. (Isla-MDTM)

### Mediation support-bridging the gap

Parents mentioned that due to the informal nature of the CSS role, CSSs often facilitated communication between families with various charities or supports in the community, and with MDT members, especially at critical times in a child’s cancer journey. This was viewed as important in enabling conversations about things that might otherwise be left unexplored:

*‘[The CSS] really facilitated the conversation being productive, both from a family and medical perspective, because [the CSS] knew the family… knew where they were coming from, [the CSS] knew what their priorities were and as a doctor I knew what my medial priorities were, but [the CSS] helped those two things to merge in a really, really positive way in a very difficult situation’.* (Daryna-MDTM)

Members of the MDT noted that the CSS’s feedback to the MDT about how things were going for the family was an important component of enhancing continuity of care and communication from the MDT:

*‘[The CSS] is really good at feeding back to us... Sometimes when you don’t hear from people you think that they’re getting on great. And that can be a bit of a red flag that people aren’t coping that well… they’re sort of families that I would refer to the community support, or the cancer support worker*. *And then [the CSS] would bring back*… *actually it’s not going great for them… maybe they could check in with you… [to say] it might be worth giving them a phone call*...’. (Karra-MDTM)

### Navigational support

Parents commented on the amount of information that they had to become acquainted with following their child’s cancer diagnosis, including available supports. The CSS supported families by voluntarily sharing information on a variety of issues ranging from who is who within the MDT, to available services available such as [names a purpose-built therapeutic centre for families affected by childhood cancer] charities, and navigating procedures involved in applying for grants, contacting charities, and services:

*‘And trying to organise then… even with the volunteers from* [names a residential camps for children with a serious illness] *to come in… just to do things with [child with cancer’s name]. [The CSS] was kind of coordinating all that in the background if [they] thought somebody needed something, if someone needed an extra little bit… the other services, information, a little bit.* [Names an Irish childhood cancer charity] *they would have been emailing us and once they had our email address and all the rest of it and offering their services. But I think the fact that the CSS was the face of it, you know it was easier than dealing with emails or phone calls’.* (Beatriz-parent)

The CSS’s willingness to proactively find relevant information for families they could not find elsewhere relieved parents of the burden of worrying about where they would access information and enabled them to concentrate on caring for their sick child. One parent spoke of the difficulty her daughter had when her eye lashes fell out because of treatment and how the CSS navigated her to YouTube clips to help and support her:

*‘… her eyebrows and her eye lashes were starting to fall out and that was freaking her out more than her hair. So, I was like, Jeez I don’t even know how to go, because I went into a salon... They were terrified thinking, oh no she got chemo that really we can’t do anything with her... I emailed… [the CSS] going is there any chance you’d have [a] clue of anything that you know to help her. And [the CSS] found… direct YouTube videos and different things... and sent them which was really helpful’.* (Olive-parent)

### Respite support for parents

CSSs provided respite support to parents which enabled them to take time for themselves to engage in self-care activities. Respite support came in many forms, tailored to individuals needs and varied from family to family. For example, within the hospital system, the CSS was described as someone who would make time to take parents for a coffee or create space for parents to go for a walk, or if needed do a small shopping errand:

*‘… even in terms of lifts… I’d be reluctant to look, ask for help… when we’ve loads of friends and everything around… When I know there’s [CSSs] people who can [help]… I think it’s really important*. *I think it’s important that they’re there for people who definitely use them*…..’. (Olive-parent)

In the community, CSSs supported and engaged the child with cancer or siblings within the home. Parents spoke of how this helped to refocus attention on siblings who may not have received enough attention over the course of their other child’s treatment. It also relieved pressure parents felt to be playing and interacting with their child/children when they were exhausted:

*‘[The CSS] might come out and you know maybe go for a walk with the girls. Or have a chat with them... I think it’ll be really good for the other two. Because you feel like you’ve ignored your other children for the last [few] months’.* (Olive-parent)

Respite support also came in the form of organising activities for the children or organising time away such as in [name of respite centre]. The fact that the CSS took the time to organise these activities also provided respite for parents, as it relieved them of the tasks associated with organising:

*‘… in terms of resources… I mean I mentioned [names a residential service]and things like that and, you know, there's things that come [the CSSs’] way, like there was a zoo trip that we were able to do, it's just kind of nice things close by. I've no capacity to be arranging things (laughs), at the moment’.* (Caroline-parent)

### Sibling support

Parents mentioned the importance of CSSs connecting with all family members, and valued warm support offered to siblings to ameliorate the challenges they encounter. Parents spoke of how the CSS made space for children to play and process their own emotions through various developmentally appropriate activities that the CSS engages in:

*‘[The CSS] comes every few weeks and oh my gosh, my children absolutely adore [them]. They love [the CSS], they talk openly with [the CSS] as if [the CSS has] been here all our lives… my little boy like I said isn’t even two so obviously he wouldn't be I suppose as involved in the stuff but he, oh my gosh the minute [the CSS] comes in he runs to [them] and he is with them all the time’.* (Kate-parent)

CSS offered opportunities for distraction for their child as well as taking a future orientation which was welcomed by parents:

*‘[The CSS] would be chatting about things that he wants to do after treatment and [the CSS] [would] be very encouraging to him, like he wants to write a book and he’d be telling [the CSS] and [the CSS] [would] be giving him tips*...’. (Ellen-parent)

## Impact on the MDT

Four subthemes representing differing impacts on the MDT were identified. These were: filling gaps of unmet needs for families; flexibility in provision of services for families; immediacy of access for families; and freeing up time for clinical component of MDT work.

### Filling the gap of unmet needs for families

MDT members noted the importance of CSSs as an auxiliary source of support which could in part meet the needs of parents. In what was described as an ‘under-resourced’ environment, the presence of the CSS role was seen as a welcome addition. The perception of MDTs was that the CSS role operated differently from other MDT team members as it was outside of the MDT’s traditional medical orientation:

*‘The role operates outside of kind of the traditional MDT, so we all have our really specific roles whereas the cancer support specialist is able to kind of provide support I don’t know I think for the families in a way that is less medicalised, less clinically orientated’*. (Olivia-MDTM)

The needs of families were emphasised by members of the MDT. Having the CSS on the ward was a relief, because it added another source of support to call on, enabling the further prioritisation of families’ multiple needs:

*‘I suppose we all had kind of almost like huge relief in us that there is somebody then that we can call when we don’t have anybody else to call….’*. (Mary-MDTM)

### Flexibility in the provision of the service for families

The flexibility and availability of CSSs at weekends was a noted benefit as many services were provided only between Monday and Friday. Weekends are also a time when there are less members of the MDT working. CSSs not being limited to a Monday to Friday, or to nine-to-five hours, ensured that support was provided if families experienced challenges or if a child was in the ICU in the evenings or over the weekend, filling this gap within services:

*‘… we see lots of challenges at weekends. And [the CSS] having that flexibility… not being stuck to a nine to four rota … is a brilliant addition I think’.* (Daryna-MDTM)

Evenings and weekends were identified as opportune times to provide additional support to families as they provide a greater opportunity to spend time with, and support, the wider family members such as siblings and other family member visitors. As there is less clinical-based activity focused on the child during these times, it also allows for a quieter, less busy, environment for families to reflect on and address some of their concerns:

*‘Whereas at least then if [the CSS] is around and has the capacity to be late and with more flexible hours, [the CSS] is catching parents at different times. If they’re juggling, like mum is here during the day, dad comes in in the evening. But he might know more about this, or he might have a worry. It’s easier to catch them when there’s a bit more leeway in [the CSS’] hours’*. (Indie-MDTM)

### Immediacy of access for families

The availability of the CSSs and their ability to provide support at the right time to familieswas welcomed by MDT members and viewed as beneficial to the care of families. This availability was partly enabled by the CSS having no waiting list:

*‘You can just kind of nab [the CSS] in the corridor and that has been the case exactly with me… I’ve referred at least three families, well not referred but have kind of brought to her attention that especially new families. They need a huge, huge amount of supports and then [the CSS] hasn’t had any waiting lists like a lot of other services’*. (Olivia-MDTM)

### Freeing up time for clinical component of MDT work

Combining clinical work with family support was spoken of as a challenge for the MDT, with clinical work often taking precedence over providing psychosocial support to families. A combination of a lack of time or specific training was noted as factors in the ability of the MDT to respond to families adequately:

*‘Because I have to go do four hundred and one other things… as much as you want to give them the time. But we’re also not hugely, we wouldn’t necessarily have a huge amount of training in how to support somebody either…Yea all of that as much as we’d love to be able to do it, there’s often the time limitations’.* (Diane-MDTM)

Members of the MDT noted how parents felt a sense of relief when the CSSS was on the ward and reported that the feedback they received from families on the impact of the CSS was overwhelmingly positive:

*‘… the feedback from families which is the most important indicator of how we are doing*

*actually has been overwhelming positive…’*. (Isla-MDTM)

The CSS’s provision of support to families thus enabled MDT members to concentrate on the clinical component of their work, while being assured that the needs of families were being met:

*‘… we might say to [the CSS] can you sit with mum or dad*, *while they run out to do something*. *And that’s been great support with us… I’m not talking about their taking over jobs from us*. *They’re actually allowing us a little bit freer to do our nursing jobs*. *And [it] is a good support… a huge support…’*. (Angelica-MDTM)

## Discussion

This study aimed to evaluate the impact of a CSSS from the perspective of families and MDT members including the CSSs. The impact of the CSSS was found to have many benefits for both families of children with cancer and the MDT. Parents spoke about informal support provided by CSSs as a source of great comfort and companionship at a particularly difficult time. Having a person available whose sole role was to spend time, be present, and provide informal psychosocial and flexible support ranging from relational to respite support made parents feel valued and helped lessen the challenges of the childhood cancer journey. As frequently found in the international literature on new informal psychosocial support roles (Bridges et al., 2003; Cain et al., 2020; Gilburt, 2016; Herber and Johnston, 2013), the flexible design of the roles was a central enabler to the integration of the CSSS.

Relational support is a keystone of effective psychosocial support (Dwarswaard et al., 2016; Robertson et al., 2024) and is an important factor in reducing parent’s stress and improving their adjustment to their child’s cancer (Melguizo-Garín et al., 2023). Many Parents welcomed having an informal source of support, as it gave them a sense of normality and enabled them to chat and discuss issues with someone separate to clinically oriented care provided by many members of the MDT. Equally, while this informal support was similar to support provided by family and friends, the CSS’s knowledge of healthcare meant that parents were not burdened by the need to explain the medical circumstances of their child’s health. Neither were parents burdened by the need to reciprocate or protect the CSSs from their pain and anguish, as they may with family or friends. The ability of the CSSs to break this information down and gradually relay it over time for improved retention was highly valued by parents. Meeting parents’ informational needs is central to being able to cope with stress (Cheng et al., 2022; Hashimoto et al., 2023; Nicklin et al., 2022). Similarly, parents reported benefits of the CSSs’ support in navigating available services and charities and assisting with filling in application and grant forms as it allowed them to focus on caring for their child. CSSs also assisted in arranging therapeutic short breaks for families in therapeutic centre (Cancer Fund for Children, 2024) where parents can relax and receive support. Respite support provided by the CSSs in people’s homes was particularly valued by parents as it allowed them to take time to engage in self-care, or rest.

CSSs’ actively supporting and engaging with children with cancer including the siblings of their child with cancer was also greatly appreciated by families. Research over the last decade shows that siblings of children with cancer patients are a vulnerable group and are at risk of social and psychological problems (Long et al., 2018). In addition, support of the community-based CSSs was valued as an important source of companionship and support for families who had returned home from hospital. Social support is one of the most important variables contributing to reducing psychological effects in parents coping with their child’s cancer (Gudmundsdottir et al., 2011, Maynard and Bennett, 2024).

MDT members spoke of relief in knowing that there was an additional informal psychosocial support provider in the form of the ward-based CSS that could help to meet the support needs of families. An MDT approach is central to the care of children with cancer and their families (Loh et al., 2023). Many MDT members spoke of their clinical duties taking up time, leaving them little time to attend to parent’s informal support needs. Expanded roles due to more complex cancer treatments (Chrapek, 2016; Lewandowaka, 2022) and understaffing are recognised as adding to the work burden of MDT members in oncology settings ( Murali and Banerjee, 2018; Seguin et al., 2022).

MDT members welcomed the CSSS as this enabled them to focus on their clinical duties while knowing that families’ informal support needs were also being met. A reduction in the work burden of MDT members has previously been identified as a benefit of the implementation of new roles providing informal psychosocial support as found in a recent scoping review (Fulham-McQuillan et al., under review). This reduction in the MDT members work burden may have benefited their wellbeing as heavy work loads are associated with burnout in MDT members in oncology settings (Guveli et al., 2015; Murali and Banerjee, 2018). The ready accessibility of the CSS on the ward was a benefit to both MDT members and families as it meant that families could get support when needed. This likely reduced unnecessary stress for families as reduced waiting times are associated with improved patient satisfaction (Abbasi-Moghaddam et al., 2019).

In addition to supplementing MDT’s provision of support during working hours, an important benefit mentioned by members of the MDT was an ability of the CSS to provide support out of hours on evenings and weekends. As many allied health hospital services operate on a 5 day model (NHS, 2013), out of hours support filled a gap in MDT’s provision of support, and was valued by staff as families often required support at these times when there previously had been no support provided. This out of hours support increased the accessibility of support for families, which is a commonly unmet need (Delemere et al., 2023).

## Limitations

When interpreting the findings, the following limitations require consideration. The findings are based on a non-probability sample of participants. Parents who participated may represent those who were most satisfied with CSSS as satisfaction could be a motivating factor in their participation. Despite reassurances of confidentiality, parents may also have refrained from negatively critiquing a service for fear that any negative comment may impact the continued funding of a service. As parents were interviewed at an emotionally challenging time in their lives, this may have influenced their recall of the CSSS impact. Had the CSSS been in place for a longer bedding-in period at the time of the study, further impacts may have become apparent, particularly in the community setting. As with all qualitative research the transferability of the findings are limited by the organisational context in which the study was conducted.

## Implications for practice

We note in this study that informal, flexible psychosocial support positively impacts families on a childhood cancer journey. Parents consistently reported in this study that they valued the variation in support offered by the CSSS from relational to respite and the whole-family perspective by the CSSS was also of great importance. The flexibility of the support offered to families by the CSSS in hospital and the community was highly appraised by the families and the MDT. The MDT reported the ready accessibility of the CSS on the ward was a benefit to both MDT members and families as it meant that families could get support when needed, the CSS also enabled the MDT to have more time for the clinical component of their work. The findings of this study have been widely communicated to relevant stakeholders. Consideration should be given on how to further develop, sustain, and integrate the CSSS into the existing services and the National Cancer Control Programme (NCCP, 2021).

## Conclusions

Through the application of a qualitative descriptive design, this study set out to examine the impact of a new CSSS on families and MDT members. This study placed the voice of families living with childhood cancer at the centre of evaluating the new service. The service was new, and its impact on families and MDT members was unknown. Evaluating the service was important to understand its future direction. Detailed analysis of the study interview transcripts highlighted the benefits of the new informal psychosocial support roles of the CSSS in providing support to the families of children with cancer in both a ward and community setting. Families received a variety of supports tailored to their specific needs. These included relational, navigational, and mediation support and respite support.

MDT members welcomed the CSSS as an auxiliary source of support for families enabling them to focus on their clinical duties. This paper provides evidence that informal support workers can respond flexibly and adaptively to the varying support needs of children and families. The work burden of MDT members while assisting in meeting the informal support needs of families of children with cancer.

Finally, the flexible design of the roles was a central enabler to the integration of the CSSS. This flexible design enabled role holders to fill gaps in the informal psychosocial support provided by the MDT during working hours, and during out of hours, and to respond flexibly and adaptively to the varying support needs of children and families.

## Supplemental Material

Supplemental Material - An evaluation of the impact of a cancer support specialist service on families of children with cancer and the multidisciplinary team in a children’s health service in Ireland Supplemental Material for An evaluation of the impact of a cancer support specialist service on families of children with cancer and the multidisciplinary team in a children’s health service in Ireland by Maryanne Murphy, Hugh Fulham-McQuillan, Agnes Higgins, and Maria Brenner in Journal of Child Health Care
