# Supplementary material for: An evaluation of the impact of a cancer support specialist service on families of children with cancer and the multidisciplinary team in a children’s health service in Ireland
Source: J Child Health Care. 2025 Jan 6;30(1):109–22. doi: 10.1177/13674935241312722 (PMC12982566; doi:10.1177/13674935241312722)
Supplement: Supplemental Material - An evaluation of the impact of a cancer support specialist service on families of children with cancer and the multidisciplinary team in a children’s health service in Ireland [file sj-pdf-1-chc-10.1177_13674935241312722.pdf]

Strategies for quality in the research

Quality criterion	Strategies
Credibility	Inclusion of different stakeholder groups (family members and clinicians)<br><br>Triangulation of data across different groups<br><br>Use of quotes to support description of themes
Dependability	In-depth description of study methods to enable replication<br><br>Using a coding framework that was inductively developed and agreed
Confirmability	Analysis completed by more than one person (MM, HFM)<br>Independently coded of data and doubled coded a random selection of interviews<br><br>Codes and themes discussed between researchers to minimise interpretative bias and achieve consensus
Reflexivity	Care was taken to work reflexively, question interpretations and assumptions. This process was helped by the fact that the research team came from different disciplinary backgrounds, including pediatric nursing, mental health and psychology
